# Supplementary material for: Pharmacists’ Knowledge, Attitudes, and Practices Toward CGRP Inhibitors in Migraine Management: A Cross-Sectional Study
Source: Healthcare (Basel). 2025 Sep 6;13(17):2231. doi: 10.3390/healthcare13172231 (PMC12427708; doi:10.3390/healthcare13172231)
Supplement: Supplementary file 1 [file healthcare-13-02231-s001.zip › supplementary S1.pdf]

You are invited to participate in a study being conducted by Dr. Fahad Alshehri, a UQU Associate Professor in the Department of Pharmacology and Toxicology. The study aims to measure Evaluating Pharmacists' Knowledge, Attitudes, and Practices Toward CGRP Inhibitors in Migraine Management in Saudi Arabia.   Participation should take approximately 10 minutes to complete the questionnaire.

PARTICIPATION

Your participation in this survey is voluntary. You may refuse to participate in the research or exit the survey at any time without penalty. In addition, you may skip any question you do not wish to answer for any reason.

BENEFITS & RISKS

You will receive no direct benefits from participating in this research study. However, your responses may help us learn more about updated statistics about Evaluating Pharmacists' Knowledge, Attitudes, and Practices Toward CGRP Inhibitors in Migraine Management in Saudi Arabia. There are no foreseeable risks involved in participating in this study other than those encountered in day-to-day life. The possible risks or discomforts of the study are minimal. You may feel a little embarrassed answering survey questions

CONFIDENTIALITY

Your survey answers will be stored initially with google forms in a password-protected electronic format.

CONTACT

If you have further questions concerning the study, contact the principal investigator at 0542166571 or by email at fsshehri@uqu.edu

ELECTRONIC CONSENT: Please select your choice below. You may print a copy of this consent form for your records. Clicking on the “Agree” button indicates that

- You have read the above information
- You voluntarily agree to participate
- You are 18 years of age or older

☐ Agree

☐ Disagree

- **Demographic**

1. Gender
   - Male
   - Female
2. Level of education
   - BSc
   - PharmD
   - Master’s
   - PhD
3. Years of experience
   - Less than 1 year
   - 1-5
   - 6-10
   - More than 10 years

4. Practice setting
   - Community
   - Hospital

5. Source of the pharmacy degree
   - Saudi university
   - Foreign university

6. Which headache condition do you most frequently encounter in your practice
   - Migraine
   - Sinus headache
   - Chronic headache
   - Tension headache
   - Do not know

7. Have you attended a course about headache/migraine management
   - Yes
   - No

8. Numbers of patients visiting your pharmacy suffering from migraines monthly
   - Less than 10
   - 11-20

- 21-30
- More than 30

- **Knowledge** (Yes/ No/ Do not know)

1. Are you familiar with Calcitonin Gene gene-related peptide (CGRP) inhibitors (e.g. Erenumab (Aimovig)) as a class of medication?
2. Do you know what are the main indications for CGRP-inhibitors?
3. Do you know how CGRP inhibitors work to treat migraines?
4. Are you aware that CGRP inhibitors have recently been added to the first-line agents in migraine prevention?
5. Are you familiar with the current guidelines or clinical recommendations in using CGRP inhibitor?
6. Is it true that CGRP inhibitors can cause an increase in blood pressure?

- **Attitude** (Strongly agree/ Agree/ Neutral/ Disagree/ strongly disagree)

1. I believe CGRP inhibitors are a better choice for migraine management than typical medications.
2. I believe the benefits of CGRP inhibitors in migraine management outweigh the potential side effects.
3. I believe there is a lack of knowledge about CGRP inhibitors among health providers.
4. I believe the injectable form of CGRP inhibitors is convenient for patients.
5. I believe that CGRP inhibitors improve the quality of life for migraine patients.

6. I believe that more research and clinical trials are needed to fully understand the benefits and possible long-term side effects of CGRP inhibitors.

- **Practice** (Strongly agree/ Agree/ Neutral/ Disagree/ strongly disagree)

1. I educate patients on the appropriate use of CGRP inhibitors.
2. I collaborate with other healthcare professionals (physicians/neurologists) for migraine management with CGRP inhibitor medications.
3. I ask about the patient's history and symptoms of their migraine type before deciding to dispense their CGRP inhibitor medication.
4. I seek additional clinical information (ADRs, DDIs, C/I) before dispensing CGRP inhibitors
5. I inform patients taking CGRP inhibitors about the possible side effects such as allergic reactions, constipation, and high blood pressure.
